# Current trends in the characterization and monitoring of vascular response to cancer therapy

**DOI:** 10.1186/s40644-024-00767-8

**Published:** 2024-10-23

**Authors:** Binita Shrestha, Noah B Stern, Annie Zhou, Andrew Dunn, Tyrone Porter

**Affiliations:** Department of Biomedical Engineering, The University of Texas at Austin, Austin, TX 78712 USA

## Abstract

Tumor vascular physiology is an important determinant of disease progression as well as the therapeutic outcome of cancer treatment. Angiogenesis or the lack of it provides crucial information about the tumor’s blood supply and therefore can be used as an index for cancer growth and progression. While standalone anti-angiogenic therapy demonstrated limited therapeutic benefits, its combination with chemotherapeutic agents improved the overall survival of cancer patients. This could be attributed to the effect of vascular normalization, a dynamic process that temporarily reverts abnormal vasculature to the normal phenotype maximizing the delivery and intratumor distribution of chemotherapeutic agents. Longitudinal monitoring of vascular changes following antiangiogenic therapy can indicate an optimal window for drug administration and estimate the potential outcome of treatment. This review primarily focuses on the status of various imaging modalities used for the longitudinal characterization of vascular changes before and after anti-angiogenic therapies and their clinical prospects.

## Background

Angiogenesis, the formation of new blood vessels, is a tightly regulated physiological process essential for tissue development and repair. It is vital for the continued growth of solid tumors as blood vessels serve as conduits for the delivery of oxygen and nutrients to support cell proliferation and the removal of waste to avoid cell toxicity. Scientists hypothesized that shutting down and possibly destroying tumor vasculature with antiangiogenic drugs would lead to cancer cell death and tumor regression. While initial studies of anti-angiogenic therapy against solid tumors produced encouraging results [1], the treatment strategy did not prove to be significantly more effective than the standard of care in clinical trials [2]. Interestingly, studies have reported that the efficacy of chemotherapeutic drugs was improved when combined with an antiangiogenic agent [3–5]. For example, the anti-VEGF antibody bevacizumab increased the survival of colorectal cancer patients by 5 months when combined with chemotherapy [6]. Whereas tumor vasculature is leaky and disorganized, scientists have speculated that anti-angiogenic agents allow blood vessels to seal properly and distribute more uniformly throughout tumors. In theory, the “normalized” vasculature would enhance the delivery of chemotherapy to a solid tumor, leading to a better therapeutic outcome. In addition, normalized vessels reprogram the tumor microenvironment (TME) by reducing tissue hypoxia and inducing an immune-supportive state [7]. Successful execution of this combinatorial/multifactorial treatment strategy would require optimizing the timing between administering antiangiogenic and cytotoxic agents, which is not trivial.

**Fig. 1:**
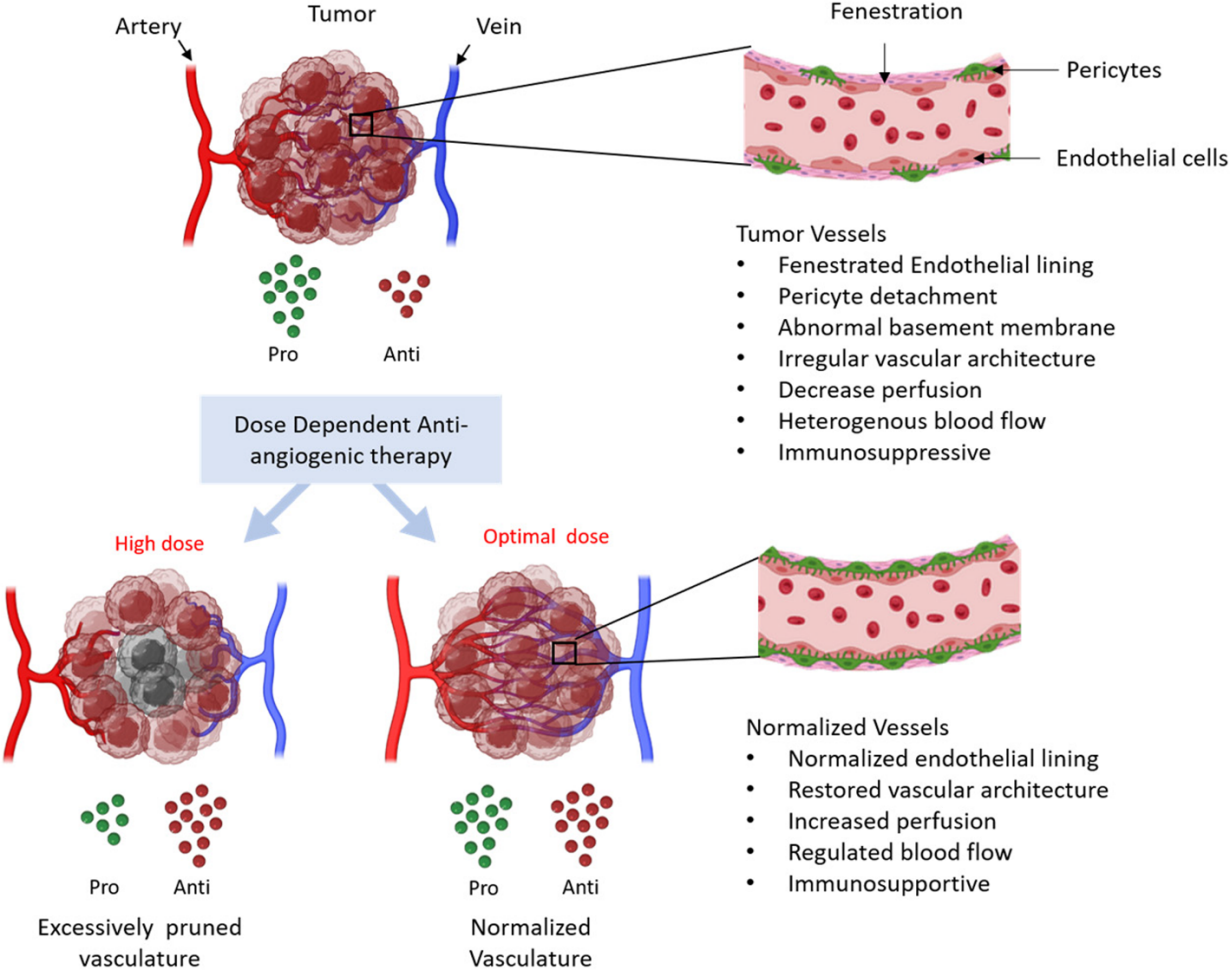
Schematic illustration of vascular normalization following anti-angiogenic therapy

Vascular normalization is a dynamic process. Research and investigations have provided critical insights into understanding this process and an optimal time frame for anticancer drug administration to maximize therapeutic efficacy. The optimal period, also known as the “normalization window”, refers to a transient period where the vessels are morphologically and functionally comparable to fully developed blood vessels (Fig. 1). Studies have shown that judicious application of antiangiogenic agents prunes immature vessels, and the remaining vasculature is less fenestrated and more uniformly distributed throughout the tumor. Ideally, cytotoxic drugs would be administered during the “normalization window” to maximize delivery and intratumor distribution. The onset and length of the normalization window, however, are time and dose-dependent and vary among different cancer types [8]. Thus, the tumor vascular response to antiangiogenic therapy must be monitored longitudinally to determine when the vessels have been normalized, at which point anticancer agents can be administered.

Various strategies such as histological staining, biomarker profiling, and imaging have been used to monitor vascular changes and evaluate the efficacy of anti-angiogenic treatment. Although histological staining is the gold standard for the detection of vascular normalization, it is not a viable option for longitudinal assessment of changes to tumor vasculature. Tissue-based or circulating molecular and cellular biomarkers can also be examined, but these require invasive procedures and provide information at the systemic level rather than localized to the tumor [9]. In the quest to longitudinally monitor vascular response to direct or indirect anti-angiogenic therapy, various imaging modalities have been explored, including intravital imaging [10, 11], MRI [12–14], CT [15, 16], PET [17, 18], ultrasound [19], and photoacoustic imaging [20–22]. Each of these imaging modalities has strength and limitation when it comes to vascular imaging (Table 1). Image-derived parameters such as tumor oxygen saturation, vessel morphology, vascular density, and blood perfusion have been used as metrics for monitoring vascular remodeling, shutdown, and normalization [23]. In this review, we will discuss and compare different imaging modalities that are used for characterizing vasculature before and after anti-angiogenic therapies and their clinical prospects followed by various standard validation methods that are currently used.

**Table 1:** Imaging Modalities Summary. Includes spatial and temporal resolutions, maximum effective depth, advantages, and disadvantages for the major modalities discussed. Components of the chart are focused on vasculature imaging purposes and are slightly different than the optimal values often reported for more general imaging procedures. For example, certain MRI and CT methodologies can give less than 100µm resolutions. Also, not included are potential super resolution methodologies which may dramatically improve or alter certain methodologies. As discussed, super resolution may play an important role in the of future ultrasound and photoacoustic vascular imaging

**Modality**	**Subtype**	**Spatial Resolution**	**Temporal Resolution**	**Depth**	**Advantages**	**Disadvantages**	**References**
**MRI**	DCE-MRI	~1-3mm^3^ voxel ~1 - 1.5mm axial	Minutes-Hours	Limitless	No ionizing radiation, limitless penetration depth, high signal to noise ratio, clinically accessible and relevant, whole-body imaging	Easily influenced by motion artifacts, very long scan times, can be high cost and various difficulties associated with siting and scheduling	[24–26]
	DSC-MRI	~2-10mm^3^ voxel ~1 - 2.5mm axial	Minutes-Hours	Limitless			[27–29]
**CT**	Perfusion-CT	5mm axial	Minutes	Limitless	Faster than MRI imaging, limitless penetration depth, whole-body imaging, widespread clinical and pre-clinical availability	High ionizing radiation, high demand on clinical systems already in use, high-end scanners which improve resolution can be expensive	[30–33]
	DECT	0.5 - 2.5mm axial	Minutes	Limitless			[34–38]
**PET**		~1-10mm axial	Minutes	Limitless	Limitless penetration depth, whole-body imaging, can directly investigate biochemical processes in the region of interest	Low resolution, high ionizing radiation, high costs	[38–42]
**OPTICAL**	OCTA	1-15µm	Seconds-Minutes	<1cm	Certain techniques can give excellent resolution, real-time imaging, low cost, wide variety in imaging techniques and fluorophores of interest	High susceptibility to artifacts, scattering, and autofluorescence in imaging region, high resolution techniques either have extremely limited imaging depths or require an invasive procedure, limited clinical application	[43–45]
	OT	~1cm	Seconds-Minutes	10cm			[46–48]
	CLE	~1-3µm	Seconds-Minutes	60-250µm			[49–53]
**ULTRASOUND**	CE-US	0.1-0.5mm	Seconds-Minutes	~12-15cm	Low cost, clinically available and prevalent, non-invasive, no ionizing radiation, real time imaging, high spatial and temporal resolution	Non-intuitive imaging that is operator dependent and difficult to interpret, limited field of view, high susceptibility to tissue artifacts and potentially limited depth	[54–57]
	DCE-US	0.2-2mm	Seconds-Minutes	~12-15cm			[57–59]
**PHOTOACOUSTIC**	PAM	OR-PAM: 1-2µm AR-PAM: 15-40µm	Seconds-Minutes	OR-PAM: 1mm AR-PAM: 3mm	Multispectral imaging allows for differentiation of endogenous and exogenous agents, no ionizing radiation, real time imaging, high spatial and temporal resolution	Limited availability, not yet clinically prevalent, large tradeoff between resolution and imaging depth, operator dependent	[60–62]
	PAT	100µm (depth dependent)	Seconds-Minutes	4-8cm			[63–66]

## Magnetic resonance imaging

MRI is one of the most frequently used imaging modalities for cancer studies and has become an important tool for researchers interested in observing the vasculature [67–69]. For vascular normalization investigations, MRI is often applied clinically for long-term studies interested in determining normalization windows, analyzing the potential of co-administered chemotherapeutics, and the overall response of the tumor microenvironment. There are a variety of MRI-based techniques that rely on contrast agents and different protocols that allow for structural, functional, and molecular imaging.

### Dynamic contrast enhanced MRI

Dynamic Contrast-Enhanced MRI (DCE-MRI) has been used to monitor vascular changes during tumor growth or regression or in response to anti-angiogenic therapy [70]*.* DCE-MRI relies on the use of gadolinium-based (Gd) paramagnetic contrast agents such as Gadobutrol and Gadodiamide that are administered systemically. For microvessels and broad neovascular imaging more specific agents like Gd-DTPA and Gd-EOB-DTPA show promise [71, 72]. DCE-MRI typically focuses on T1-weighted images as Gd is known to shorten the T1 and T2 relaxation times [73]. Images are acquired before, during, and after the contrast material has flowed into and through the tissue of interest. The acquired MR signal is used to generate a time-intensity curve (TIC) that corresponds to the arrival of the contrast agent represented in enhancement values. Pharmacokinetic models, such as the Tofts model [74, 75], are used to analyze the contrast agent TIC derived via DCE-MRI to obtain physiological properties such as vessel permeability, blood flow, vessel surface area product, and composition of interstitial space [76–80]. Generation of these increasingly complex pharmacokinetic models is an active area of research and has been used for identifying and subtyping tumor tissue [81–83], lesion characterization [84], and studies of the vasculature [85–88]. Dozens of parameters can be obtained from these pharmacokinetic models, but *K*_*trans*_*, v*_*e*_*,* and *k*_*ep*_ are most often investigated. *K*_*trans*_, or the volume transfer coefficient, is a constant that describes molecular exchange from blood plasma to extravascular space. It is a function of both permeability and vessel surface area [89, 90]. v_e_ represents the volume of extravascular space per volume of tissue in which contrast agents accumulate [91]. *k*_*ep*_ is a rate constant describing the exchange rate from extravascular space back to blood plasma. These parameters have been directly related to important physiological values implicated in vascular normalization like vessel density, permeability, and size. As such, DCE-MRI is commonly used to investigate the extent of normalization in the tumor microenvironment.

**Fig. 2:**
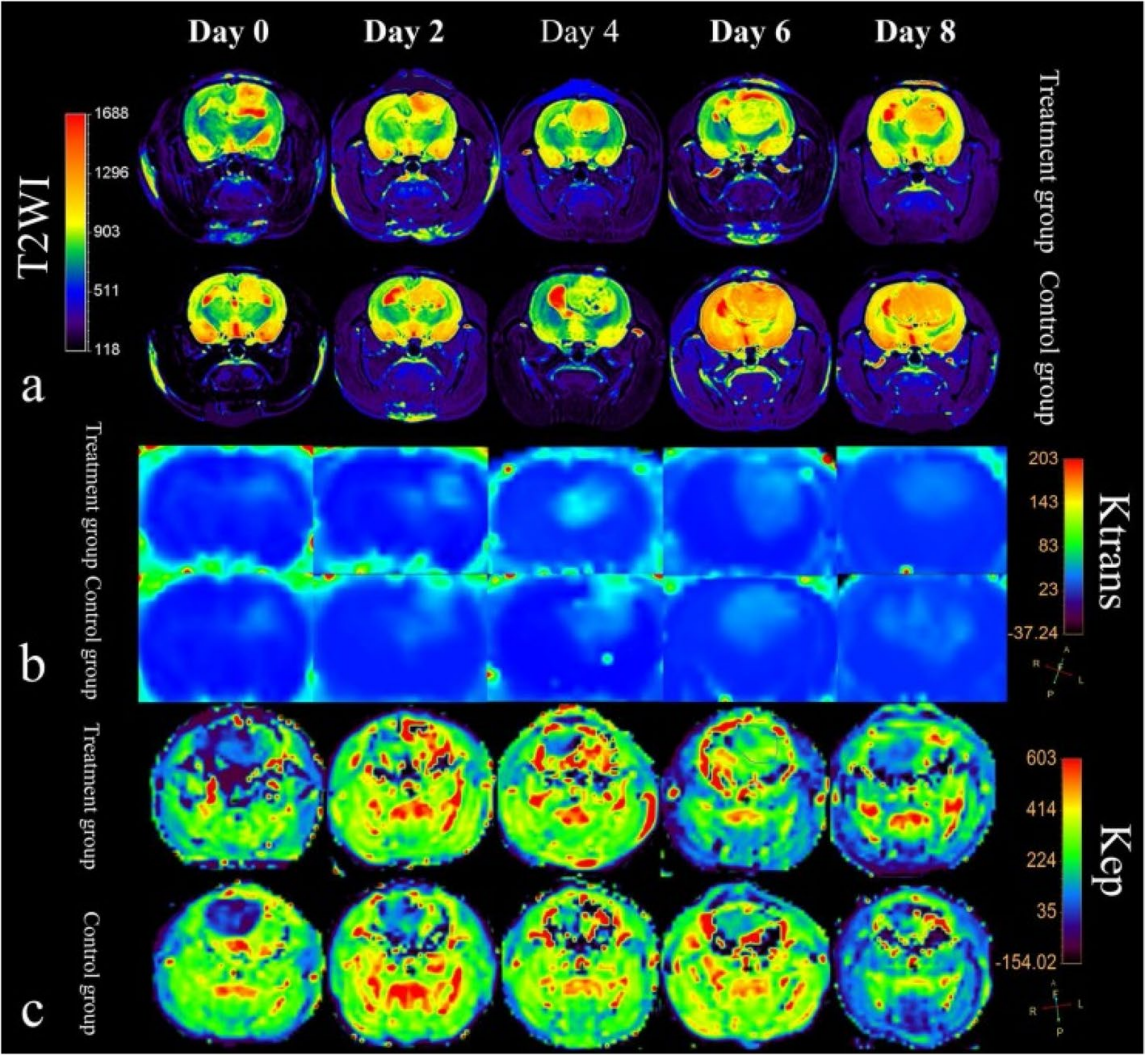
**a** T2-weighted images of rat brains on days 0 to 8 for treatment (top) and control (bottom) groups. Treatment groups received a single dose of anti-angiogenic bevacizumab on day 0. Shown by regions of higher (more red) T2WI signal, control tumors had higher levels of peritumoral edema than bevacizumab treated tumors on days 2 and 4. Outside of the normalization window, days 6 and 8, edema is restored as tumor growth continues. **b** and (**c**) color maps for DCE-MRI *k*_*trans*_ and *k*_*ep*_ around tumors from day 0 to day 8. *K*_*trans*_ and *k*_*ep*_ values are most significantly increased when compared to controls on days 2 and 4, before reducing on days 6 and 8. Increased levels of *k*_*trans*_ and *k*_*ep*_ are a sign of increased perfusion and a more normalized vasculature. Crucially, these subtle and transient increases help identify this “vascular normalization window” where therapies may be more effective due to this increased perfusion

Recently, multiple groups have linked *K*_*trans*_ to vascular function and have attempted to show correlations between *K*_*trans*_ and markers of angiogenesis like microvessel density (MVD) [92–94]. *K*_*trans*_ has been commonly used in vasculature normalization studies as shown by several recent investigations with bevacizumab [95–97]. Chen and Lu et al. showed a significant normalization response and presented evidence for the benefits of a long duration pretreatment to enhance the efficacy of potentially co-administered chemotherapeutics [98]. Pishko et. al similarly showed substantial vascular normalization response, slowed tumor growth, limited increase in edema, and importantly a significant decrease in tumor vessel permeability [96]. Yang et. al were also able to elucidate the relatively short normalization window (1 to 2 days) and showed a significant correlation between values of *K*_*trans*_ and *k*_*ep*_ with levels of perfusion. As shown in Fig. 2, from Yang and collaborators, the highest levels of *K*_*trans*_ and *k*_*ep*_ are observed between days 2 and 4 after a single dose of bevacizumab in treatment groups [97]. Increased *K*_*trans*_ and *k*_*ep*_ are representative of a more normalized vasculature system in these tumors.In another recent study investigating sorafenib and infigratinib, significant *K*_*trans*_ changes were associated with diminished tumor growth, mitigation of tumor hypoxia, and a more normal cellular microenvironment [99]. Studies also have reported that decreased levels of *K*_*trans*_ as early as 1 day after anti-angiogenic treatment is indicative of positive response in glioma patients [100–102].

While these pharmacokinetic models have shown clinical value, they are limited by their complexity and potentially time-consuming implementation. To avoid these hurdles, certain semi-quantitative values can be obtained in near real-time directly from the time-course of the contrast agent. The common metrics include the initial area under the curve (iAUC), the slope of the steepest portion of the time-course signal, and time-to-peak enhancement. These values can be used individually or in combination with aforementioned quantitative parameters, as shown by Chen and Lu et. al who associated K_*trans*_ values with significant decreases in iAUC and slope values [98]. In another study of tumor response to bevacizumab, iAUC was implicated as a strong prognostic marker of positive patient response prior to normalization [103]. iAUC, time-to-peak enhancement, and K_*trans*_ were all shown to correlate with vascular parameters obtained from optical coherence tomography [104]. While not specific to vascular normalization, Zeng and Zhang *et. al*. demonstrated slope, time-to-peak enhancement, and several other semi-quantitative parameters that had significant clinical value in monitoring chemotherapy response [105].

### Dynamic susceptibility contrast MRI

Dynamic Susceptibility Contrast MRI (DSC-MRI) also uses gadolinium-based contrast agents but differs from DCE-MRI in that the technique capitalizes on the local inhomogeneities produced from differences in magnetic susceptibility during the first pass of the contrast agent through the body. Large gradients in susceptibility between vasculature and tissue contribute to significantly reduced T2 relaxation times, meaning T2-weighted images show significant contrast enhancement [12, 79, 106–108]. As these gradients are formed due to the localized susceptibility differences between the paramagnetic contrast agent and diamagnetic tissue, the generated signal and overall contrast enhancement are heavily dependent on vessel architecture. The tight junctions prevalent in the blood-brain-barrier make these differences even more pronounced and contributes to the prevalence of DSC-MRI in brain imaging applications [109, 110]. Similar to DCE-MRI, kinetic models can be created based on tracer or indicator dilution theory [111] and allow for the generation of quantitative maps of cerebral blood volume (CBV), cerebral blood flow (CBF), and mean transit time (MTT).

CBV is the fraction of the overall volume occupied by blood and is the simplest to obtain as it is proportional to the integral of the relaxation rate curve. CBF refers directly to the rate of blood delivery to the brain tissue. To estimate the CBF, it is necessary to mathematically deconvolute measures of agent concentration in the tissue of interest and vasculature. MTT is the average amount of time it takes for a single contrast agent to pass through the vasculature. Based on the central volume principle, MTT = CBV/CBF [112]. These blood flow specific parameters coupled with the use of DSC-MRI use for brain imaging, make it an effective choice for investigating vascular normalization in glioma. Relative CBV has been implicated as a potential predictor of biological changes and overall glioma patient response to bevacizumab [113–115] In a recent retroactive study, CBV and CBF were associated with reduced tumor angiogenesis and improved overall survival in bevacizumab-treated patients but had mixed results for the predictive capabilities of the technique [116]. Cho et al. went even further and not only investigated CBV as an indicator of bevacizumab’s effect but also connected DSC measurements to a potentially improved patient response when also blocking CCL2, a chemokine implicated in the differentiation of tumor-associated macrophages [117]. Interestingly, another recent study of bevacizumab in recurrent glioblastoma showed that despite normalization, overall tumor oxygenation worsened [118].

### Other MRI-based approaches for monitoring vascular response

DCE-MRI and DSC-MRI are the most common MRI techniques used for investigating vascular normalization, but other MRI techniques have been successfully employed. Intravoxel incoherent motion MRI (IVIM-MRI), which does not require a contrast agent and offers the ability to separate the pure diffusion and perfusion characteristics of water molecules, has the potential to become an effective tool for monitoring normalization [119]. Recently, IVIM-MRI perfusion and diffusion parameters were correlated with pericyte coverage and histological parameters associated with vascular normalization [120, 121]. Blood oxygen level-dependent MRI (BOLD-MRI), which relies on the magnetic susceptibility differences between oxygenated and deoxygenated hemoglobin, also is an attractive option for measuring response to anti-angiogenic therapy without an exogenous contrast agent [122, 123]. Liang et al. combined DCE-MRI-derived *K*_*trans*_ values with BOLD-MRI measurements to support the idea that bevacizumab can improve pericyte coverage and increase perfusion [124]. Ma et al. used both IVIM-MRI and BOLD-MRI as a means to investigate a variety of parameters associated with the tumor microenvironment following a combined anti-angiogenic and hypoxia-activated drug treatment regimen [125].

MRI is a powerful medical imaging tool with a wide variety of applications due to its high spatial resolution and lack of ionizing radiation. These advantages in conjunction with the many types of imaging protocols and the ability to co-register functional, molecular, and topographical images make MRI an attractive imaging tool for vascular normalization research. Parameters like *K*_*trans*_ and CBF can be directly related to fundamental changes in extravasation, perfusion, diffusion, and overall normalization, which give unique insights to clinicians and researchers alike. A quantitative metric of normalization, the “vascular normalization index”, has been proposed that combines multiple parameters including the MR-derived K_trans_ and CBV to provide a metric of normalization that may be a predictor of normalization success and patient survival [126]. However, MRI is commonly associated with long scanning times, high cost, and high sensitivity to motion artifacts. Critically, common MR techniques lack the spatial resolution needed to image vessel at the capillary level. Apart from these common issues, some normalization studies can be hindered by the potentially complex pharmacokinetic models required for analysis and the indirect relationship between commonly investigated parameters and changes observed in the tumor microenvironment. With the availability and the requisite expertise, MRI stands as one of the most useful imaging modalities for vascular normalization studies.

## Computed tomography

Computed Tomography (CT) uses x-rays as the basis for image construction. These x-rays attenuate differently as they pass through tissues with varying density in the body, leading to the characteristic contrast difference between skeletal and soft tissue. Iodine-based commercial contrast agents like Iomeprol and Iohexol are often used for soft tissue imaging [127]. X-ray traces are combined and back-projected to form individual 2D tissue slices that can be cycled through and stacked to form a 3D image of the patient [128–131]. The attenuation visualized in these images is directly related to the local concentration of contrast agents in the tissue [132]. Without any further processing, these images already provide considerable structural information, but by monitoring changes in concentration over time it is possible to determine values associated with blood flow and perfusion. This mix of available structural and functional information makes CT a viable option for monitoring vascular response and normalization.

### Perfusion computed tomography

Perfusion CT is a form of dynamic contrast-enhanced CT that is commonly employed for vascular imaging and analysis. Similar to the time-course concentration profiles previously described for MR imaging, perfusion CT relies on the creation of time-attenuation curves. Typically, a set of baseline CT images is collected and then compared to a series of images obtained over time after an intravenous bolus injection of contrast agent. From these images, it is possible to relate signal attenuation to the concentration of contrast agent in circulating blood over time [133–136]. These time-attenuation curves can be analyzed or parameters like the slope of the steepest portion, the area under the curve (AUC), mean transit time (MTT), time to peak enhancement, and overall peak enhancement [137], or can be fit to quantitative pharmacokinetic models. Most commonly, the Johnson and Wilson [138] or Patlak [139] kinetic models are employed. These models give a few values that can be more directly related to anatomical and physiological features of the vasculature in the tissue or organ of interest. Regional blood flow (BF) and blood volume (BV) provide values for the flow rate and total volume of blood in the imaging region. The extraction fraction (EF) describes the fraction of contrast agent that is transferred to the extravascular space during a single passage. The permeability surface area product (PS) describes the total diffusion-based flux across the capillaries, similar to the *K*_*trans*_ value obtained via MR imaging. Also available through further processing are maps for arterial perfusion (AP) and perfusion index (PI). Overall, this collection of parameters gives insight into oxygen delivery, vessel leakage, and vessel density, and these parameters could be monitored longitudinally to identify the vascular normalization window.

Due to its widespread availability and reproducibility, perfusion-CT has been used extensively in clinical applications of anti-angiogenic agents [140–144]. Perfusion-CT’s ability to elucidate parameters directly related to vasculature function makes it a powerful prognostic tool for patients [145–147]. Zou *et. al* were able to correlate a variety of factors including AP and PI to MVD and VEGF expression levels, allowing for improved differentiation of benign and malignant lung lesions [147]. It has been shown that after a single cycle of treatment, BF and BV could help predict patient response to anti-angiogenic agents [148]. Jiang et. al. not only showed a significant reduction in BF, BV, and PS and significant increases in MTT after anti-angiogenic treatment but also connected these parameters back to patient response [149]. Kambadakone et al. demonstrated that perfusion-CT could be used to detect a positive tumor response to bevacizumab and radiotherapy by capturing significant reductions in BF and BV, which correlated with changes in tumor MVD [142]. Heist et al. provided further support for a vascular normalization window by showing that a standard dose of bevacizumab significantly reduced BF, BV, and PS, indicative of an overall drop in perfusion. Instead of completely impairing vasculature, greater survival benefits may be related to improved perfusion through tumor vasculature [143]. PS also has been shown to correlate with values obtained for *K*_*trans*_, making perfusion-CT a potentially attractive option in situations where MRI is unavailable [150].

### Dual energy computed tomography

Dual Energy CT (DECT), also known as Spectral CT, is a recent advancement on conventional CT that relies on gathering data from two or more peak energies following the injection of a contrast agent, typically iodine. This is done by tracking attenuation from two different tube voltages, typically one low (~70 keV) and one high (~140 keV). DECT uses advanced sensors and software to combine these separate acquisitions creating richer and more complex images [151–154]. Along with the slope of the attenuation curves, the most common method for analyzing DECT images is to track the iodine concentration (IC) obtained from iodine maps. As the iodine maps show the degree of vascularization in the imaged tissue, the volumetric uptake of iodine can be representative of tissue perfusion. DECT also offers substantial benefits over conventional CT angiography with superior temporal resolution [155, 156] lower radiation dose [157], and easier differentiation of vascular tissue from the background [158].

While not entirely interchangeable, DECT is a viable and potentially superior alternative to conventional contrast-enhanced CT. Several groups have reported a good correlation between metrics like BV, BF, and MTT from perfusion CT with ICs from DECT in a variety of cancer types [159–162]. Additionally, DECT was shown to provide a significant improvement over conventional imaging and classification techniques specifically when evaluating the anti-angiogenic response to bevacizumab [163]. In another bevacizumab-focused study, Han et al. reported that the slope of the energy spectrum curve and the IC over time correlated positively with changes in tumor size as well as VEGF and HIF-1a expression levels [164]. Separate studies into four different tyrosine kinase inhibitors provided evidence that DECT can be employed for early prediction of patient response. More specifically, studies into axitinib [165], sunitinib [166], regorafenib [167], and sorafenib [168] demonstrated that normalized IC values could be predictive of a favorable response and that the parameters related to IC, such as volumetric iodine-uptake, can be more sensitive in the early detection than other CT methods.

### Micro-computed tomography

Perfusion-CT and DECT are the most commonly employed types of CT for monitoring vascular response to therapy due to their clinical availability, speed, and high resolution, but micro-CT has been applied in some research settings outside of clinical applications. Briefly, micro-CT uses very similar technology to conventional CT, but at a much smaller scale and with enhanced resolution [169]. This means micro-CT cannot be used clinically but does have a wide array of applications for small animal models and ex-vivo samples [170] including monitoring vascularization and the effects of anti-angiogenic treatment [171]. Gu et. al capitalized on the enhanced resolution of micro-CT to demonstrate a significant drop in relative vessel density for vessels smaller than 50 microns [172] following anti-angiogenic therapy while Hutchenreuther et. al were able to use micro-CT to investigate the role of cancer-associated fibroblasts and the CCN2 pathway in tumor neovascularization [173]. Micro-CT is often characterized by the impressive 3D reconstructions it can generate, and while it cannot be used clinically these information-rich reconstructions make micro-CT a valuable modality for investigating micro-vasculature.

Overall, CT is a vital imaging tool across the medical field that has structural, functional, and molecular imaging capabilities. CT is available in most clinical settings, is repeatable, and provides high resolution with short scanning times. With a multitude of methods and scanners, there are many ways in which CT can be utilized to characterize the response of vasculature to anti-angiogenic therapies. Qualitative parameters associated with contrast agent delivery and more quantitative parameters related to blood flow and perfusion can be obtained and tracked well with physiologic responses in patients. One of the main drawbacks of CT is the use of ionizing radiation, which can limit the frequency of imaging trials and is potentially harmful to patients. CT images also are sometimes susceptible to a variety of artifacts that can obscure tissues of interest. Nonetheless, CT remains one of the most commonly employed tools for investigating the tumor microenvironment and is a very good option for monitoring vascular normalization.

## Positron emission tomography imaging

Positron Emission Tomography (PET) is used for sensitive and quantitative molecular imaging of cells and tissues. PET imaging is based on the detection of the radiation emitted from a radiolabeled tracer administered systemically [174]. The radiolabeled tracers are designed to bind with specific biomolecules involved in disease progression or treatment response. To date, various PET tracers have been produced for the assessment of various diseases [175–177], particularly in cancer [178, 179]. For example, 2-deoxy-2-[18F]-fluoro-D-glucose (18F-FDG PET) can be used to detect tumors based on their elevated glucose metabolism [180, 181], whereas 3′-deoxy-3′-18F-fluorothymidine (18F-FLT) PET detects tumors based on increased DNA replication [182].

Targeted contrast agents also have been engineered for PET-based molecular imaging of tumor angiogenesis. For example, α_v_β_3_ and α_v_β_5_ integrins are heterodimeric transmembrane glycoproteins overexpressed in newly formed vessels. Arginine-glycine-aspartate (RGD) specifically binds to α_v_β_3_ integrins and has been conjugated to radiotracers for PET-based molecular imaging of angiogenesis [183–185]. The dimeric or polymeric RGD peptides demonstrate superior binding affinity for α_v_β_3_ in comparison to their monomeric counterparts [186]. Thus, RGD peptides labeled with various isotopes such as ^18^F, ^68^Ga, and ^64^Cu have been evaluated as tracers for α_v_β_3_ integrin PET imaging. ^18^F RGD-K5 in particular has been used in clinical studies to image angiogenesis on various types of cancer including breast cancer, lung cancer, head and neck cancer, and lymphoma. Radiolabeled RGD has been used to study the relationship between angiogenesis and tumor blood flow [187]. However, laborious labeling procedures and low yields have hindered the clinical translation of PET tracers. Synthesis of improved RGD tracers for PET imaging is continuously being pursued. Guo *et. al* reported a one-step RGD labeling procedure to prepare [18F] AIF-NOTA-PRGD2 and compared its kinetic parameters with well-established RGD tracers, [^18^F]FPPRG and [^68^Ga]Ga-NOTA-PRGD [188]. [^18^F] AIF-NOTA-PRGD2 demonstrated comparable binding affinity to the aforementioned radiotracers; however, the agent showed high uptake which may be due to nonspecific accumulation and a lower clearance rate [188]. [^18^F] AIF-NOTA-PRGD2 also has been used in a clinical trial to evaluate the activity of apatinib, an antiangiogenic agent [189].

Recently in a similar study, aminopeptidase N receptor (APN/CD13) was used as a biomarker of angiogenesis [190]. APN/CD13 is highly expressed in angiogenic blood vessels and correlates well with cancer progression. Asparagine-glycine-arginine tripeptide sequence (NGR) has high selectivity for APN/CD13 [191] and has been investigated as a tracer for PET imaging where scientists introduced a lactosamine derivative to NGR that binds with galectin-3, also overexpressed in certain cancer types, to develop a dual-targeting tracer [190].

**Fig. 3:**
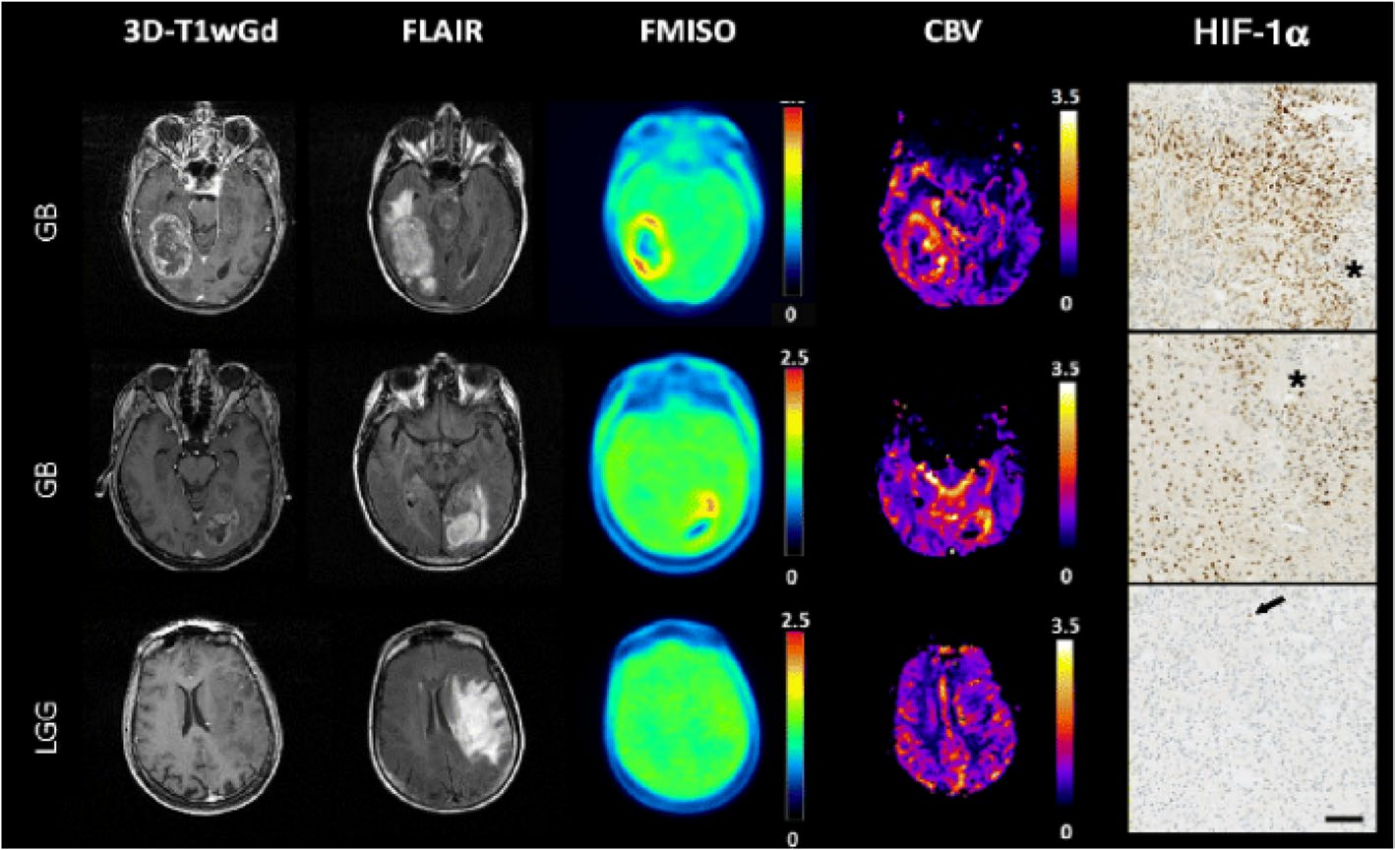
Three dimensional T1 weighted gadolinium (3D-T1wGd) injected MRI image, Fluid attenuated inversion recovery (FLAIR) image. F-MISO, cerebral blood volume map, and HIF-α immunostaining images of two patients with glioblastoma (GB) and low-grade glioma (LGG). GB patients showed enhanced contrast and necrosis in the MRI image, hyperintensity in the FLAIR image, high F-MISO uptake and higher CBV intensity whereas no contrast enhancement or F-MISO uptake was observed in patients with LGG. These results were further validated with HIF-α immunostaining in which high nuclear expression of HIF-α was observed in GB shown by asterisks and minimal to no HIF-α expression in LGG. Scare bar: 500 µm [191]

The PET tracer [18F]-FMISO binds to the macromolecules in hypoxic tissue enabling detection by PET. [18F]-FMISO uptake by hypoxic tissue has been verified by biological markers on a molecular level (Fig. 3) [192]. In one study, hypoxia was used as a surrogate parameter, to identify and trace vascular normalization using an 18F-misonidazole [18F]-FMISO [193]. Hypoxia collectively stems from increased interstitial pressure, edema, and altered metabolism, as a result of abnormal vasculature. Therefore, hypoxia has been used as an indirect measure of vascular normalization. In this study, dovitinib, a multi-targeted tyrosine kinase inhibitor was used for anti-angiogenic treatment followed by chemotherapeutic drugs. Tumor uptake of FMISO corresponds to hypoxia and was a proxy for the identification and tracking of vascular normalization. In a phase II randomized trial, [18F]-FMISO was used to evaluate the benefits of a novel multi-tyrosine kinase inhibitor, nintedanib, combined with the chemotherapeutic drug, paclitaxel [194]. PET-based detection of [18F]-FMISO was used to evaluate interstitial oxygenation levels as a measure of vascular normalization and to determine whether to continue administering nintedanib.

Prostate-specific membrane antigen (PSMA), a protein highly overexpressed on prostate cancer endothelial cells, is another biomarker used in PSMA-PET imaging for staging and restaging prostate cancer [195]. Although first characterized in the prostate, PSMA is also expressed in the neovascular endothelium of non-prostate solid tumors while there is little to no expression in normal vascular endothelial cells. This variable expression makes PSMA a potential molecular target for diagnosis and therapy in other malignancies such as breast cancer [196, 197], colorectal cancer [198, 199], renal cancer [200, 201], glioblastoma multiform [202, 203], thyroid cancer [204, 205], pancreatic cancer [206], hepatocellular cancer [207] and others [195, 208, 209]. Increased PSMA expression can be used as a surrogate for increased tumor angiogenesis in cancer, which is associated with poor patient prognosis. For example, PSMA upregulation is associated with lower overall survival, higher tumor size and cell proliferation in breast cancer, and higher histologic grades in non-small cell lung cancer. PSMA has been used for determining the aggressiveness of various types of cancer [210] and for differentiating malignant tumors from benign [206] based on changes in tumor vasculature.

^18^Fluoride (^18^F) and ^68^Gallium (^68^Ga) are common isotopes used to label PSMA antigen ligands [211, 212]. (^18^F) F-DCFPyL [213] and Glu-NH-CO-NH-Lys-(Ahx) also known as 68Ga(HBED-CC) or ^68^Ga-PSMA-11 gozetotide are FDA-approved diagnostic radiotracers for PSMA-PET imaging for prostate cancer [214, 215]. Recent studies have used ^68^Ga-PSMA-11 and (^18^F) F-DCFPyL to diagnose or stage non-prostate cancers by leveraging neovascular PSMA expression. For example, ^68^Ga-PSMA-11 was used to detect and stage renal carcinoma. In this study, PSMA-PET enabled the detection of sub-centimeter lesions which is challenging with other standard imaging systems like CT and MRI. Likewise, (^18^F) F-DCFPyL radiotracer was used to localize metastatic differentiated thyroid cancer by detecting angiogenesis within the tumor [216].

^68^Ga isotope is widely used for labeling PSMA ligands for PSMA-PET imaging. However, ^18^F may be preferable due to shorter positron range, higher positron yield, and economic practicality [217, 218]. Furthermore, the accumulation of ^68^Ga in the urinary bladder could mask the presence of a lesion in the prostate or prostatic bed resulting in a false negative diagnosis.

One of the major limitations of PSMA-PET is the non-specific expression of PSMA in normal tissues such as salivary glands, renal tubules, small intestines, the brain, liver, spleen, etc. Moderate to intense uptake of PSMA radiotracers in various organs has been observed. Retaining these tracers in different organs makes interpreting PET images challenging and may hinder the clinical diagnostic outcome. Additionally, histopathological studies indicate significant inter and intra-heterogeneity in PSMA expression among various cancer types. In parts, significant discrepancies may be attributed to different techniques and products used in the analysis [208]. Due to the lack of a standardized quantification method, it is difficult to determine PSMA availability for PSMA-PET imaging.

PET imaging can be performed as static or dynamic scanning. Static PET imaging provides a spatial map of regional tracer concentration whereas dynamic PET scanning provides both temporal and spatial information of the tracer [219]. Visual inspection and standardized uptake value (SUV) are used regularly for image interpretation in a static PET imaging system in a clinical setting. SUV is a semiquantitative approach and is the ratio of the radiotracer concentration in a region of interest and the injected activity divided by normalization factors such as body weight, body surface area, and lean body mass [220]. Static PET was used to monitor vascular normalization following treatment by tyrosine-kinase inhibitor, dovitinib [193]. The SUV quantification method does not require arterial cannulation and has a shorter scanning time, making it a cheaper and more patient-friendly alternative to dynamic PET imaging. However, SUV approximation does suffer from significant variability introduced by slight differences in experimental procedure [220]. Dynamic PET enables the measurement of tracer kinetics and provides mean tissue radioactivity as a function of time. The compartmental modeling approach is considered the gold standard in PET imaging to quantify the kinetics of tracers. However, it requires complex dynamic data acquisition and arterial blood sampling. Other analysis methods used for oncological PET imaging include the Logan plot and the Patlak plot. The Logan and Patlak plots are widely accepted methods for reversible and non-reversible tracer kinetic analysis. Although, dynamic PET provides superior information in comparison to conventional static PET, its clinical application is hampered due to duration of the protocol and limited axial field of view (FOV). However, new generation PET/CT equipped with an extended FOV and sophisticated software packages are expected to significantly contribute in oncological diagnostics [221].

To summarize, PET is highly sensitive for molecular and functional imaging of vasculature. PET Imaging allows for the detection of isotopes in target tissues at picomolar concentrations. However, the anatomical resolution of PET is limited to 1-2 mm^3^ in small animal imaging systems and 4-8 mm^3^ in clinical imaging systems. This requires PET to be aided by CT or MRI to improve anatomical resolution [222]. Furthermore, PET imaging is highly dependent on the binding affinity of tracers and the expression of target receptors in blood vessels. While the toxicity of radiotracer is mitigated by the use of picomolar concentrations, the low-yield labeling procedure is the major challenge in the synthesis of radiotracers.

## Optical imaging

Optical imaging refers to a broad family of techniques that uses light and leverages the properties of photons to investigate tissues and cells. Optical imaging is minimally or non-invasive and can achieve sub-cellular resolution. Various optical imaging methods such as optical coherence tomography angiography, optical tomography, and confocal laser endoscopy can be used to image vasculature in clinical settings for diagnosing an assortment of diseases and monitoring vascular response to treatment.

### Optical coherence tomography angiography

Optical coherence tomography angiography (OCTA) is a noninvasive method for imaging blood flow in the retina and choroid. OCTA shares similarities with optical coherence tomography (OCT) imaging. Both methods operate on the principle of interferometry, generating images by measuring differences in the amplitude and delay of light that has been reflected or backscattered by the sample [223]. While OCT directly measures structural information, OCTA detects vasculature by the motion contrast created by circulating blood cells. The movement of blood cells in vasculature produces shifting signals in successive scans of the same region and is distinguished from the unchanging signals produced by static features [223, 224].

OCTA has been used to successfully identify abnormal vasculature in the squamous epithelium of the cornea and conjunctiva, choroids, and skin [225–228]. OCTA was used in a study to evaluate the effectiveness of topical treatments for patients diagnosed with ocular surface squamous neoplasia (OSSN) and were presenting tumors [225]. OCTA images were taken to assess tumor vasculature at three time-points: before treatment, at mid-treatment, and post-treatment during tumor resolution. OCTA allowed for changes in vessel area density in the tumor to be monitored with treatment. In another study, OCTA was used to evaluate the vascular structure of choroidal neovascularization (CNV) in patients that had been treated with multiple injections of VEGF. This allowed for the identification of common vascular features in CNV [226]. While OCTA is primarily used for imaging eyes, preliminary studies have been completed for imaging capillaries in skin [227, 228]. Deegan *et. al*. used OCTA to demonstrate structural differences in microvasculature among numerous skin conditions and in comparison to healthy skin [227].

A major advantage of OCTA is that it produces depth-resolved images and can visualize microvasculature (i.e., capillary networks). OCTA also benefits from not requiring the use of contrast dye. However, some drawbacks include limited quantitative information about blood flow, the inability to determine alterations in vascular permeability or detect vascular leakage, and image artifacts that potential may lead to misinterpretation of the images with respect to vascular biology [224]. Furthermore, OCTA has limited imaging depth, and therefore, is restricted to superficial tissues.

### Optical tomography

Optical Tomography (OT) is an optical imaging technique that measures the scattering of near-infrared (NIR) diffused light in tissue. NIR source wavelengths are in the range of 700-900 nm. Concentrations of oxygenated and deoxygenated hemoglobin can be determined from the measured optical absorption, which provides information on the vascularity of a region [229]. Hemoglobin concentrations can indicate tumor angiogenesis, a marker of tumor growth or metastasis, as well as tumor hypoxia, which can correlate to tumor response to therapeutic treatment [230–232]. OT has high temporal resolution but suffers from limited spatial resolution. Thus, an additional imaging modality, such as ultrasound (US) or magnetic resonance imaging (MRI), often times is used in conjunction with OT to provide additional spatial information.

With localization and structural information provided through US or MRI, OT has been used in clinical studies to characterize, diagnose, and monitor the hemoglobin and oxygen levels of cancerous and noncancerous breast lesions [230, 231, 233]. Zhu *et. al*. studied the hemoglobin distribution and blood oxygen saturation levels in US-visible breast lesions clinically and demonstrated the utility of OT with US to monitor tumor vascular response to chemotherapy [8, 9, 230, 231]. Another study of breast lesion used MR images to provide structural information that were overlaid with OT images during analysis. Blood oxygenation and hemoglobin concentration values were calculated to investigate the oxy- and deoxyhemoglobin content of the lesions and used to characterize the different lesion types [233]. In a separate study, OT was used to detect the difference in perfusion-related parameters within days following treatment with an anti-angiogenic agent in responding and non-responding cell lines [234]. By detecting and monitoring hemoglobin and oxygen levels to glean vascular density information, OT offers metrics with the potential to help predict clinical outcomes in breast cancer patients.

Albeit high temporal resolution, the lack of sufficient spatial resolution limits its application as a standalone imaging technique and requires OT to be combined with other imaging modalities.

### Confocal laser endoscopy

Confocal laser endoscopy (CLE) operates on the principles of confocal microscopy, which is a fluorescence imaging technique that illuminates tissue with a low-power laser and collects reflected, in-focus light through a pinhole spatial filter. Currently, there are two types of CLE. One is probe-based, consisting of confocal miniprobes in the accessory channel of an endoscope. The other is endoscope-based, where a confocal scanner is built into the tip of the endoscope [235].

CLE has been used to identify irregular vasculature associated with carcinoma in the colon, liver, bladder, stomach, and esophagus [236–239]. When using CLE for examining the tumor mucosa, De Palma *et. al*. found that blood vessels in tumor tissues were more dilated and tortuous, with higher branching, leakage, and abnormal blood flow compared to normal tissue [236]. A study that used CLE for examining gastric cancerous mucosa found that undifferentiated gastric cancers exhibited hypovascularity, with abnormal, short branch vessels, while differentiated gastric cancers were hypervascular and tortuous with varied shapes and diameters among the microvessels [237]. The same study also investigated esophageal carcinomas with CLE and found dilated and/or abnormally tortuous vessels in the mucosa with unusual variability in shape and size [237]. A study of lesions in the liver identified vascular patterns associated with neoplasia with 86% accuracy [238]. A CLE study of the urothelial carcinoma associated distorted vasculature with high-grade cancer [239]. Across these studies, CLE has demonstrated diagnostic potential through vascular imaging of various carcinomas.

Although, CLE has been primarily used to histological evaluation of gastrointestinal system, Confocal laser endomicroscopy has been investigated to evaluated vascular networks in fresh biopsies of malignant colorectal tissue. This technique demonstrate potential application in clinical setting for monitoring anti-angiogenic therapy [240]. Advantages of CLE include having a high spatial resolution, allowing for the detailed evaluation of vascular structure at cellular level. Some drawbacks, however, include limited imaging depth and the need for a contrast agent (i.e., fluorescent dye such as fluorescein) to be administered topically or intravenously [235, 241].

### Multi-photon microscopy

Multi-photon microscopy (MPM) is an optical imaging technique that can be used to visualize vasculature *in vivo*. MPM uses one or more long-wavelength coherent laser source to excite a fluorophore using two or more photons, each photon carrying a fraction of the energy that is required for single-photon excitation.

**Fig. 4:**
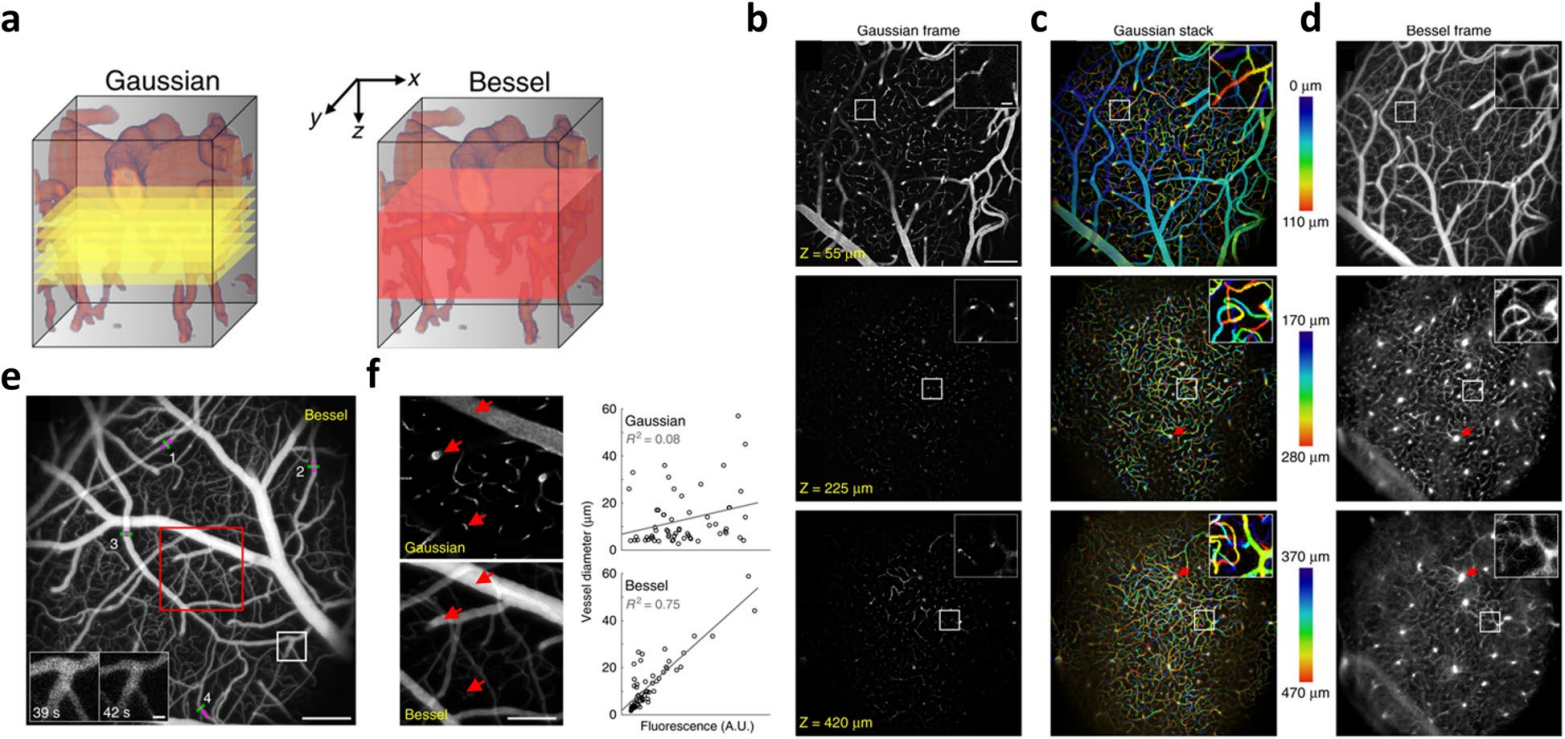
**a** Schematic illustration of Gaussian and Bessel volumetric TPLSM. Gaussian volumetric imaging is acquired using 2D images taken at multiple Z-positions whereas Bessel volumetric image is acquired in a single frame within the volume defined by the 2D scanning area. **b** Gaussian images of vasculature acquired with Texas Red labeled dextran at different depth (Z= 55μm, 225 μm and 420 μm) (**c**) Depth-dependent color-coded Gaussian image stacks within 0-110 μm, 170 – 280 μm and 30 – 470 μm. **d** A 100 -110 μm thick Bessel frame acquired within the volume of (**c**). **e** A grayscale vasculature image acquired with Bessel TPLSM. Insets represents the zoomed-in views of the whiteboxed region showing changes in vessel size at 39 s and 42 s. **f** Gaussian frame at Z= 50 μm and Bessel images of the red-boxed region in (**e**) captured at different times. Red arrows indicates large, medium and small vessel for comparison between Gaussian and Bessel TPLSM [249]

MPM has been used to investigate morphometric details of excised blood vessel walls [242]. MPM has been used to study tumor vasculature in conjunction with surrounding cells to create a wholistic image of the tumor microenvironment [243, 244]. Brown *et. al*. used MPM to study the relationship between VEGF and angiogenesis in tumors, quantify tumor blood flow with red blood cell velocity measurements in tumor vessels, and quantify the permeability of tumor vessels *in vivo* [243]. Recent advances in MPM for tumor vascular imaging include the design and exploration of new dyes, such as AIE luminogen (BTPETQ), which was shown to improve imaging depth and signal-to-background ratio, and Pluronic® fluorescent nano micelles, which were found capable of imaging leaky tumor vessels [245, 246]. Other particles have been created for imaging and photodynamic therapy (PDT), which allows for two-photon imaging of vasculature concurrent PDT treatment [247, 248]. Additionally, multi-photon luminescence imaging of gold nanoparticles as a contrast agent has been used to investigate monitor vascular permeability in mouse brain in vivo [249]. MPM recently has also been used to track gold nanoparticles within vasculature, unleashing the potential to obtain real time structural and functional information of vasculature *in vivo*, which is essential to monitoring vascular changes during and post treatment [249]. Recently two-photon laser scanning microscopy (TPLSM) equipped with Bessel focus module was used to capture volumetric hemodynamics in live mice at a spatial and temporal resolution that can be used to obtain structural and functional information of blood vessels (Fig. 4) [250]. With such extensive work done in animals *in vivo*, MPM shows potential as a future technique for clinical evaluation of tumor vasculature.

MPM is advantageous over confocal imaging in that the excitation is focused to a spot, with minimal background fluorescence created or collected, which reduces the risk of phototoxicity. The use of long excitation wavelengths also allows for deeper imaging of tissue. A major disadvantage of MPM is the risk of thermal damage to the subject during imaging, although this can be avoided with careful and proper use of the laser at appropriate power levels [243, 251]. Similar to other optical imaging methods, MPM also suffers from limited imaging depth.

## Ultrasound imaging

Ultrasound (US) is a clinical imaging modality that has been investigated for the assessment of various diseases such as rheumatoid arthritis [252], inflammatory bowel disease [253], cardiovascular disease [254, 255], and various types of cancer [256–258]. In general, ultrasound is a non-invasive and safe diagnostic imaging modality that offers many advantages including affordability, accessibility, and shorter scan time. In addition, ultrasound provides superior soft tissue contrast and relatively deep imaging depth. B-mode US imaging is the most commonly used technique and serves as the basis for ultrasound imaging. This involves the transmission of ultrasound pulses and the collection of reflected echoes by the ultrasound transducer. As the sound waves propagate through tissue, a fraction of the transmitted waves is reflected depending on the acoustic impedance of the tissues. The varying amplitude of US echoes collected by the transducer is then translated to pixel intensity to allow visualization and quantification of anatomical structures. In a clinical setting, B-mode imaging is used to identify cysts, lesions, tumors, and other structural or functional anomalies. While regular B-mode imaging is limited to identifying anatomical features, contrast-enhanced dynamic US imaging enables functional imaging of vasculature. Contrast-enhanced US imaging has been utilized to monitor changes in tumor perfusion and blood flow during tumor progression or antiangiogenic therapy.

### Contrast-enhanced US imaging

Contrast-enhanced US imaging (CEUS) has dramatically broadened the scope of US imaging through the application of contrast agents. CEUS has demonstrated higher detection sensitivity via signal enhancement and high temporal resolution. Microbubbles are the most common contrast agent used for CEUS. Microbubbles are core-shell particles with a gas core and either lipid or albumin shells that are typically 1-4 µm in diameter. There are many commercially available microbubbles such as Optison, Definity, Sono Vue, and USphere, a few of which are already FDA-approved. The gas core, which is usually biologically inert, determines the stability of microbubbles, for example, perfluorocarbon gas cores generate sufficiently stable microbubbles [259]. Microbubbles have a high echogenicity and can generate a strong acoustic signal in an acoustic field as a result of radial oscillation, which exceeds the amplitude of the acoustic waves reflected by tissue interfaces [260]. The stability and signal amplitude of the microbubbles depends on the properties of the gas core, the overall size of the bubbles, the nature of the surrounding medium, and the frequency and power of the incident ultrasound echo. Hemodynamically, microbubbles are identical to red blood cells. At the steady state, the enhancement in acoustic intensity is directly proportional with the microvascular blood volume. For perfusion imaging, a high-amplitude ultrasound pulse is transmitted to burst microbubbles flowing through a tissue volume of interest. The tissue volume is replenished with circulating microbubbles, and the rate of increase in echo intensity provides important information with respect to the extent of vascular perfusion in the tissue of interest blood velocity, the product of two provides microvascular perfusion measurement [260, 261].

**Fig. 5:**
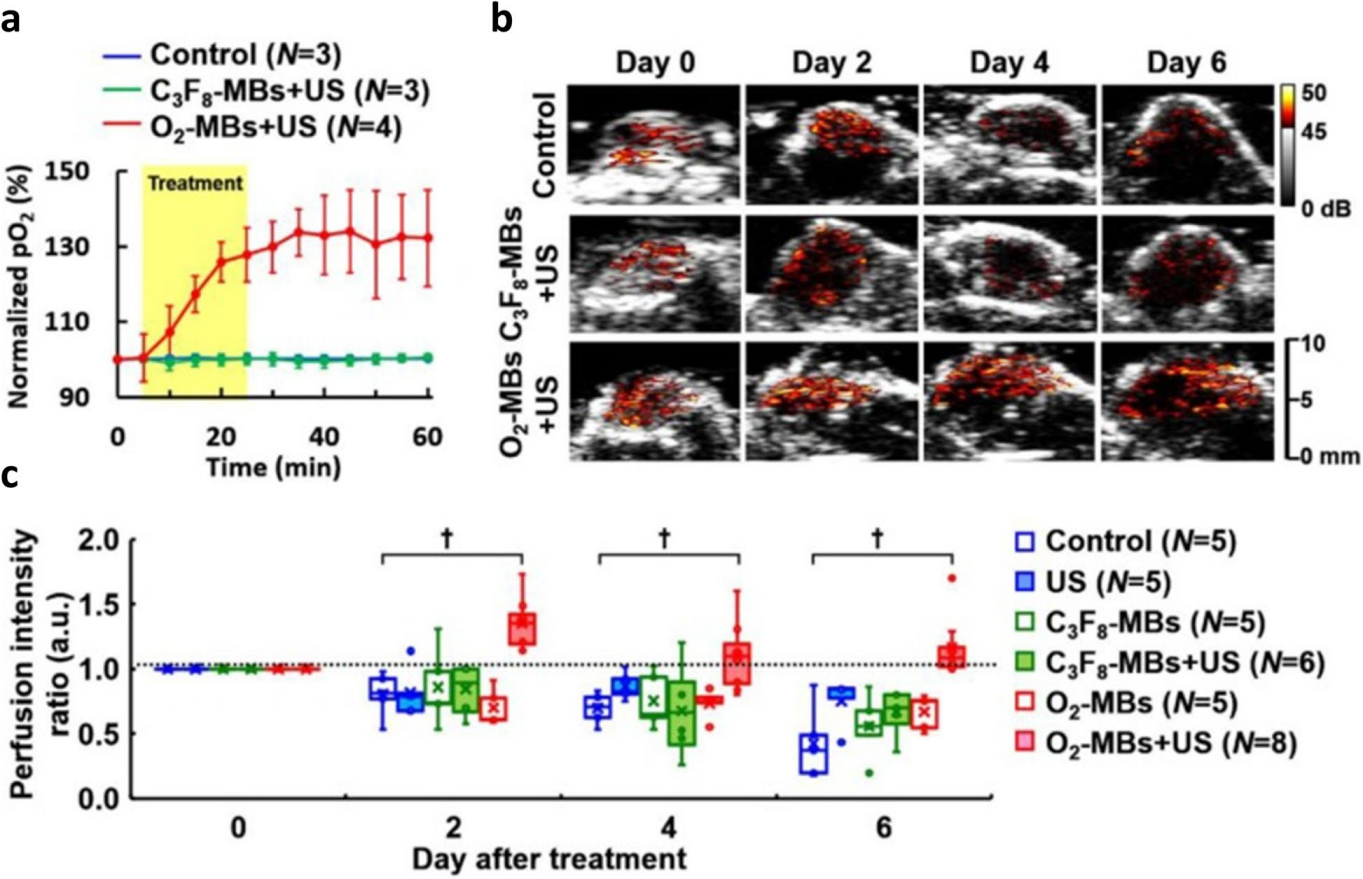
Enhanced tumor perfusion through oxygen delivery. **a** Normalized partial oxygen pressure in the control group tumor, and tumor treated with C3 F8 and O2 microbubbles. **b** Change in blood perfusion traced at different time points using US contrast imaging. **c** Graphical representation of perfusion intensity at different time points [264]

CEUS has been used to evaluate the therapeutic outcome of cancer treatment by monitoring tumor angiogenesis [262]. Moreover, microbubbles can be targeted to tumor vasculature for localized monitoring of vascular changes [263]. In one recent study, CEUS was used to trace oxygen-microbubble (O_2_-MB) induced vascular normalization [19]. In this study, microbubbles were fabricated with various volume ratios of oxygen and perfluoropropane (C_3_F_8_). The O_2_-MBs were used as an oxygen delivery vehicle and C_3_F_8_ microbubbles were used as a contrast agent to evaluate tumor perfusion. An increase in tumor oxygenation achieved through external US stimulation led to inhibition of Hypoxia-inducible factor 1—alpha (HIF-1α) expression (Fig. 5a), which further reduced VEGF transcription and mitigated tumor angiogenesis [264]. O_2_-MBs may induce vascular normalization without decreasing vascular density unlike anti-angiogenic agents, which may result in vascular regression [19]. The pre- and post-C_3_F_8_-MB injection images were quantified to evaluate tumor perfusion (Fig. 5b). Following treatment with O_2_-MBs, C_3_F_8_-MBs were infused via an injection pump with a velocity of 0.3 ml/h to maintain the vascular concentration of C_3_F_8_-MBs during ultrasound contrast imaging. The administration of O_2_-MBs led to higher perfusion, which resulted in better drug delivery to established tumors in comparison to control and C_3_F_8_-MBs.

### Dynamic contrast-enhanced ultrasound

Dynamic contrast-enhanced US (DCE-US) is a multiparametric functional imaging technique that can be used for quantitative imaging of tumor perfusion [265]. DCE-US imaging has further advanced US diagnosis through digitized quantification of contrast uptake on recorded video data. In general, for DCE-US, a high dose of ultrasound contrast agent is injected followed by an immediate flush with normal saline. The images are recorded for 3 minutes following injection and analyzed to evaluate treatment response. DCE-US allows more accurate characterization of tumor vascularity than conventional B-mode US and has been successfully used for angiogenesis quantification in cancer patients [266, 267]. DCE-US is used to evaluate quantitatively blood flow and blood volume as measures of perfusion [268, 269]. Parameters such as mean transit time, peak intensity, time to peak contrast intensity, wash in time, washout time, and slope of the contrast wash are collectively used to evaluate therapeutic outcome [262]. Additionally, the contrast-enhanced ultrasound intensity can be plotted as a function of time and the AUC can be calculated. Studies have shown that a significant drop in AUC reflects a detectable change in tumor perfusion [268, 270] and thus, can be used as an image-based metric for monitoring treatment outcomes in cancer [271–273].

DCE-US provides superior temporal resolution which enables real-time microvessel perfusion and repeatable short examinations. DCE-US offers crucial insights into the heterogeneity of tumor vascularization that correlates well with standard histological measures; however, vessel structural features are difficult to resolve with this technique due to the limited spatial resolution [274]. Another major challenge with DCE-US imaging is the limited circulation time of microbubbles. The circulation half-life of microbubbles following intravenous injection is less than 7 min [275] which limits the image acquisition time window. Nevertheless, DCE-US serve as a viable image-based option for determining the “normalized window” for anti-cancer therapies.

### Acoustic angiography

Acoustic angiography is a CEUS imaging approach that is used for high-resolution microvascular imaging [276]. Acoustic angiography is performed using dual-frequency transducers with non-overlapping bandwidths. The “transmit” transducer has an operating frequency close to the resonance frequency of the microbubble contrast agents and transmits pulses to drive nonlinear bubble oscillations. The “receive” transducer has an operating frequency that is a harmonic of the transmit transducer and receives superharmonic emissions radiated by the microbubbles [277]. Because tissue is not a strong source of nonlinear acoustic emissions, acoustic angiography images can be constructed with a high contrast-to-tissue ratio. Using a high frequency transducer as the receiver allows for construction of high resolution images. This imaging modality provides high-resolution three-dimensional maps of microvasculature with little tissue background and can resolve 100-200 µm diameter vessels up to to a depth of approximately 150 mm [278, 279]. Acoustic angiography has been used as a diagnostic tool to identify tumors and monitor changes in vessel morphology and densities [279, 280]. For example, the modality has been used to detect changes in microvasculature density following radiotherapy of cancer [281]. Recently, arterial labeling US subtraction angiography has been developed, utilizing PFC nanodroplets for imaging single blood vessels [282]. Acoustic angiography can be combined with DCE-US imaging to monitor changes in the structure and function of microvasculature during cancer treatment and potentially predict clinical outcome [283].

Acoustic angiography offers a cost-efficient and non-invasive tool for monitoring morphological changes in tumor vasculature indicative of a positive tumor response to therapy. For example, Rojas et al. demonstrated that acoustic angiography could detect changes in vascular density following antiangiogenic and Notch inhibition therapies one week prior to a reduction in tumor volume [284]. The combination of acoustic angiography with functional ultrasound imaging such as DCE-US could be an effective approach to evaluate and monitor vascular normalization. The major challenge in acoustic angiography, however, is the availability of dual-frequency transducers that can both transmit low-frequency signals and be able to detect harmonics at higher frequencies. There are ongoing efforts to meet this need by developing new dual-frequency transducers [285–287].

### Doppler US imaging

Doppler US imaging methods are well-established tools for real-time quantitative analysis of blood flow and are heavily used in clinical diagnosis. Tumor blood flow can be abnormally slow and fluctuate with time, and in some cases can be stagnant or reverse direction [288]. Quantitative measurements of blood flow and directionality, along with vessel structural details, can provide crucial insight into the response of tumor vasculature to antiangiogenic therapy. There are multiple formats for Doppler sonography, including spectral Doppler, color Doppler, and power Doppler. Each format will be described briefly along with their advantages and disadvantages with respect to interrogating and displaying blood flow.

There are two versions of spectral Doppler: Continuous-wave Doppler (CWD) and Pulsed-wave Doppler (PWD). In CWD, ultrasound waves are transmitted and backscatter from flowing cells are received continuously and used to estimate the velocity along a user-defined path. CWD is ideal for analyzing high velocity blood flow, which is observed frequently in vascular pathologies (i.e. atherosclerosis). A major disadvantage of CWD is the inability to determine where the velocities are estimated along the user-defined path. Unlike CWD, PWD sends short pulses (<30 cycles) of sound repeatedly and alternates between emission and reception of ultrasound signal. PWD offers gating in which users can define a small area where the Doppler shifts are recorded and allow the estimation of blood velocity at a specific location. However, estimates of blood velocity are less accurate with PWD for high velocity flow and at greater distances from the transducer. It is important to note that spectral Doppler plots flow velocity as a function of time separate from an ultrasound image. Other Doppler modalities, such as color and power Doppler, create spatial maps of blood flow that can be combined with B-mode images of the tissue and vasculature.

Color Doppler is the color-coded visualization of average velocity and direction in the region of interest defined by the user. In color Doppler, blood flowing away from the transducer is represented as blue while blood flowing towards the transducer is represented as red. However, similar to PWD, when a Nyquist limit is reached (*f*=PRF/2), Doppler shifts cannot determine accurately the flow direction and velocity. Compared to color Doppler, power Doppler provides greater detail about blood flow and is particularly useful for analyzing vessels at greater depths and with low-velocity flow. However, power Doppler does not provide information on the directionality of blood flow. 3D power Doppler represents a more reliable and reproducible approach for the assessment of tumor vasculature [289]. Donnelly *et al.* demonstrated that power Doppler US imaging could be used to assess quantitatively changes in tumor vascularity and blood flow after treatment with radiation and/or a molecular therapeutic (Donnelly et al Radiology 2001). More recently, ultrafast Doppler tomography was used for quantitative assessment of tumor angiogenesis at different stages of cancer development [290]. Another group employed 3D power Doppler aided with Virtual Organ Computer-aided Analysis to characterize flow in tumor blood vessels and to determine the diagnostic threshold for accurate assessment of tumor and therapeutic measures [291]. The quantification and real time information on blood flow along with vessel structural details can provide crucial information to determine the normalization window for optimal cancer treatment.

Originally, ultrasound scatter from red blood cells was used to calculate the Doppler shift. However, red blood cells are poor scatterers, which made estimating flow velocities in deep-seated vessels with Doppler ultrasound challenging. Ultrasound contrast agents (UCA) can be used to overcome this shortcoming and enable characterization of hemodynamics in tumor vasculature with Doppler US imaging. Krix *et al*. demonstrated that contrast-enhanced power Doppler US imaging could be used to monitor changes in blood volume and mean flow velocity in tumors during growth and during anti-VEGF2 treatment [292]. Unfortunately, Doppler US imaging operated at clinical frequencies (<10 MHz) cannot resolve flow in vessels smaller than a few hundred microns, which are typically the first to respond to antiangiogenic agents. This shortcoming can be addressed with high-frequency ultrasound transducers, thus enabling characterization of blood flow in small vessels in solid tumors with Doppler US imaging [293–295]. Jugold *et al*. demonstrated that three dimensional 30-MHz Doppler ultrasound could be used to assess the response of tumor vessels with small diameters to anti-angiogenic therapy in murine tumor xenografts [296]. However, the imaging depth of high-frequency ultrasound is severely limited due to acoustic attenuation. To address this limitation, researchers are developing novel signal processing schemes for super resolution ultrasound imaging without sacrificing imaging depth, as described in the following section.

Another limitation of clinical Doppler is low sensitivity to slow blood flow, which may occur during anti-angiogenetic therapy. To address this challenge, Ultrasound microvessel imaging (UMI) has been developed. UMI employs high frame rate plane wave imaging and Eigen base clutter filters to improve the signal-to-noise ratio in Doppler sonography, thus improve the ability to analyze slow-velocity blood flow [297, 298].

### Super-resolution US imaging

One of the inherent limitations of conventional diagnostic US modalities is the inability to image the microvasculature due to acoustic diffraction-limited spatial resolution. To evaluate and monitor vascular response to treatment, the ability to visualize and investigate both structural and functional aspects of microvasculature is critically important. This has fueled the development of super resolution US imaging. Super-resolution US imaging is based on centroid localization and tracking of individual microbubbles, which enables sub-diffraction US imaging at depths up to a few centimeters [299]. Christen *et. al* reported super-resolved images and velocity maps from an unmodified clinical US system utilizing postprocessing localization algorithms [299]. They were able to resolve vessels in US images with a diameter of 10 µm at imaging depths exceeding 1 cm. Researchers have demonstrated that super resolution US imaging can visualize structural changes in tumor microvasculature in response to bevacizumab [300]. Additional studies are needed to assess the correlation between changes in microvasculature detected with SR US imaging and tumor response to treatment.

Two major factors that limit the resolution for resolving microvasculature are localization uncertainties and localization densities. Moreover, the ultrasound contrast agent concentration is kept low in order to localize and track individual microbubbles. The scattered ultrasound signal from individual microbubbles is relatively weak, but this can be overcome by acquiring and combining thousands of images of circulating microbubbles. Unfortunately, the time required to acquire and process thousands of images to achieve a sufficient signal-to-noise ratio makes super resolution image construction in real time extremely difficult. Researchers are exploring various approaches for reducing the number of images or the time required for processing in order to make real-time SR ultrasound imaging achievable [301–303]. Super resolution US would undoubtedly advance diagnostic capability of US to a next level. However, super resolution US is still in its nascent stage limited by physical and computational attributes.

## Photoacoustic imaging

Photoacoustic imaging (PAI) also known as optoacoustic imaging is a hybrid technology that integrates optical and acoustic aspects of imaging technology to enable higher spatial resolution at greater imaging depth. Optical imaging such as a confocal microscope, multi-photon microscope, and optical coherence tomography provided superior spatial resolution; however, the imaging depth is limited to a few millimeters in highly scattering tissues and therefore can be restricted to the assessment of superficial vasculature. On the other hand, ultrasound imaging, though limited in spatial resolution, provides greater imaging depth. PAI has emerged as a promising modality to address the traditional limitation of both optical and acoustic imaging. It is a non-ionizing, safe and noninvasive imaging approach and therefore has gained rapid momentum in clinical diagnostic imaging [304]. Photoacoustic imaging leverages the acoustic component to overcome the fundamental penetration limit of optical imaging. The photoacoustic effect is the physical phenomenon describing the generation of acoustic waves following laser irradiation and photon absorption. When biological tissues are irradiated with a laser, the photons are absorbed and converted into heat resulting in thermoelastic expansion of tissues. The resulting transient thermal expansion of tissue generates acoustic waves proportional to the optical absorption of endogenous chromophores such as hemoglobin, lipid, and melanin. These acoustic waves are detected by a transducer and are processed to generate images. The endogenous chromophores, hemoglobin in particular, interest the scientific community at large as they enable visualization and characterization of vasculature without utilizing additional contrast agents. Additionally, photoacoustic imaging of hemoglobin can be used to quantify oxygen saturation in blood vessels.

As such, PA imaging can serve as a non-invasive label-free approach for the assessment of microvasculature through various structural or geometric parameters such as vessel diameter, density, tortuosity, and fractal dimensions [305]. These attributes of PA imaging make it an attractive alternative to determine, evaluate and monitor vascular normalization following antiangiogenic therapies or other therapeutic measures. The major modalities of photoacoustic imaging used in the assessment of vasculature include Photoacoustic Microscopy, Photoacoustic Tomography, and Photoacoustic endoscopy/Intravital imaging. Photoacoustic endoscopy is primarily used to visualize lipid deposits in atherosclerotic plaques in arteries. Photoacoustic endoscopy is beyond the scope of this article and therefore will not be discussed.

### Photoacoustic microscopy (PAM)

Photoacoustic microscopy, similar to optical microscopy systems, provides a high spatial resolution of 1-2 µm at a comparable imaging depth of 0.2-1 mm. The extinction coefficient for oxyhemoglobin (HbO_2_) and deoxyhemoglobin (Hb) varies with wavelength, and PAI systems leverage these differing spectral characteristics to estimate oxygen saturation in blood vessels and the surrounding tissue through spectral unmixing algorithms [306]. PAM has been used to resolve structural and functional features of vasculature, such as morphology, oxygen saturation, and blood flow in the brain with an intact skull [307] or through a cranial window. PAM can be classified into (i) optical-resolution photoacoustic microscopy (OR-PAM), and (ii) acoustic resolution photoacoustic microscopy (AR-PAM).

#### OR-PAM

OR-PAM has been successfully used to resolve structural, functional, molecular, and genetic characteristics at the cellular and subcellular level through both endogenous and exogenous contrast agents [308]. OR-PAM uses a highly focused laser beam for excitation and a focused or unfocused ultrasound transducer for detection of acoustic emissions. OR-PAM is performed by point scanning the overlapping foci of the transmitting laser and the receiving ultrasound transducer. The optical focal spot size of the focused laser determines the lateral resolution whereas axial resolution is determined by the transducer’s bandwidth. The optoacoustic beam combiner technology is commonly used for *in vivo* microvascular imaging to tightly focus light providing a lateral resolution below 5 µm at an imaging depth on the order of a millimeter [309, 310]. The lateral resolution, however, can be reduced at the expense of imaging depth [308]. Various design and computational strategies are being pursued to improve sensitivity and imaging depth, [311–314] and for preclinical imaging [315]. For instance, OR-PAM has been used to evaluate hemodynamics in the brain of anesthetized [316] or awake mice [307, 317].

Zhou *et. al* recently monitored longitudinal vascular changes and identified a normalization window in a mouse ear prostate cancer xenograft following anti-angiogenic therapy [318]. The vessel diameter, density, and tortuosity were successfully quantified in OR-PAM images to assess tumor development. Following treatment with DC101, a significant decrease in vessel diameter and tortuosity was observed whereas the overall vessel density remained unaffected. These observations were further validated with histological analysis. It was also shown that the normalized vasculature gave rise to an increased accumulation and homogeneous distribution of the chemotherapeutic agent in the tumor. They reported gradual vascular normalization until day 5 following DC101 treatment, after which the normalized vessels were eliminated. Another study demonstrated the feasibility of OR-PAM in the diagnosis of ovarian cancer [319]. They reported differences in vessel volume, length, and the number of segments in benign vs malignant excised ovarian and fallopian tube specimens. Although they were able to distinguish malignancy in the excised ovary, adopting this approach for *in vivo* assessment would prove challenging due to the limited imaging depth of OR-PAM.

#### AR-PAM

Unlike OR-PAM, AR-PAM utilizes a lightly focused laser beam for illumination and a focused transducer for detection. Similar to OR-PAM, the axial resolution of AR-PAM is determined by the transducer’s bandwidth. However, the lateral resolution is determined by the central frequency and numerical aperture of the transducer [320]. In AR-PAM, both lateral resolution and imaging depth can be tuned based on the frequency of the transducers [321]. For instance, a higher frequency transducer provides better lateral resolution but with limited imaging depth due to higher acoustic attenuation in biological tissue. A lateral resolution of 45 µm and an imaging depth of 3mm have been reported with a 50 MHz transducer. On the other hand, lower frequency transducers can be utilized for imaging at deeper locations but with lateral resolution trade-offs [320, 322].

Real-time PAM imaging, however, has been hindered by slow scanning speeds of the laser focus [307]. The implementation of different scanning technology such as MEMS scanners, and voice coils have improved PAM imaging [323]. Wang *et. al* demonstrated real-time imaging of single-flowing red blood cells delivering oxygen with a temporal resolution of milliseconds [324]. Although, very powerful, PAM might be limited in its ability to investigate structural and functional aspects of vasculature to accurately determine normalized window or vascular response to therapy in deep seated cancers.

### Photoacoustic mesoscopy and macroscopy

Unlike PAM, which has limited imaging depth, Photoacoustic mesoscopy and maroscopy enable deep tissue imaging [282]. Mesoscopy can be described as a bridge between PAM and PA macroscopy [325]. Mesoscopy refers to imaging depth up to 5mm with the resolution ranging from few microns to tens of microns, whereas macroscopy refers to the imaging depth beyond 5mm with depth dependent resolution ranging from tens of micros to a few hundred microns [282]. The imaging performance of these classes of photoacoustic imaging relies on ultrasound sensors and dectectors geometry as well as image receonstruction algorithms.

### Photoacoustic mesoscopy

Photoacoustic mesoscopy or raster scanning optoacoustic mesoscopy (RSOM) is a modified concept of AR-PAM that utilizes wide-field optical illumination and single tightly focused high center frequency transducers [326]. RSOM provides superior resolution, in the range of 15 – 40 µm with the imaging depth of 2 mm, enough to distinguish vessels of different sizes and resolve vascular response to cancer therapy [327]. For example, Raster-scanning optoacoustic angiography was used to characterize and quantify neoangiogenesis in colon cancer models [328]. In one study, RSOM was able to image vessels with 20 µm diameter as well as identified the small changes within the vascular network of tumor [329].

### Photoacoustic tomography

Photoacoustic tomography (PAT) is the traditional form of PAI macroscopy that has been extensively investigated for qualitative and quantitative imaging and evaluation of deeper vasculature. Similar to PAM, PAT capitalizes on the strong absorbance of hemoglobin to image the blood vessel network and does not require additional exogenous contrast agents. This capability of PAT has opened up multiple avenues for diagnostic research, particularly in diseases such as cancer and cardiovascular disease, which are characterized by significant changes in functional and morphological aspects of the vasculature at increasing depth. As such, one of the primary applications of PAT is detecting and monitoring vasculature changes during disease progression and assessing the role of the vasculature in disease development. For example, Lao *et. al* investigated the morphological changes in tumor vasculature over 20 days following the subcutaneous inoculation of breast cancer tumor cells in a small animal model [330]. PAT also can be used as an imaging tool for evaluating the therapeutic efficacy of a treatment regimen. PAT has been used to monitor transient vascular normalization and to determine the optimal window for delivering cancer therapeutics. In another study, PAT was used to assess the functional measurement of ovarian tumor response to trebananib. PAT demonstrated both vessel regression and normalized vessels following anti-angiogenic treatment which was further validated by serum biomarker profiling of angiopoietin 1 [331]. Besides hemoglobin, other intrinsic absorbers such as melanin and lipids have been explored for the diagnosis of various diseases including melanoma and atherosclerosis respectively.

Multispectral optoacoustic tomography (MSOT) has further expanded the application of photoacoustic imaging, particularly in the functional assessment of vasculature. In addition to resolving blood vessels as small as 100 µm at depths approaching 1 cm, MSOT can assess blood oxygen saturation levels [332]. In MSOT, tissues are illuminated at multiple wavelengths and absorbance spectra are collected. A spectral unmixing algorithm is then used to resolve oxygenated and deoxygenated hemoglobin as an individual absorber and hence differentiate arteries from veins. Oxygen saturation provides perfusion hemodynamics that is crucial to identifying or monitoring a diseased state [333]. For example, low oxygenation (hypoxia) may indicate a tumor [334] or a lower degree of oxygen saturation may indicate vascular diseases.

Various customized and commercial PAT systems built using a wide array of detectors are being continuously designed for improving different aspects of vascular imaging in a variety of research and clinical applications. For instance, Taruttis *et. al* investigated a handheld probe with a concave array for clinical assessment of major blood vessels and microvasculature [332]. In another study, Matsumoto used a hemispherical detector array for 3D imaging of human palmar vessels [335]. An alternative light source such as a light-emitting diode has been explored for point-of-care PAT imaging [336]. Other commercial systems are available for vascular imaging in a selected ROI [337] or whole-body imaging of small animals [338, 339]. Although PA imaging provides better spatial resolution at greater imaging depth, whole-body photoacoustic imaging currently is only applicable to small animal models. The clinical application of PA imaging, though extensively investigated, remains limited to relatively superficial areas of the body unless conducted intraoperatively. Moreover, the utility of PAT in human brain vasculature imaging is severely limited mainly due to a lack of sensitivity to functional changes, imaging speed, penetration depth, and skull-induced aberrations [340]. Nevertheless, continuous efforts are being pursued on instrumentation as well as technical aspects to improve upon existing PA imaging systems for functional imaging of the brain [340–342] and other clinical and preclinical applications [343, 344].

While significant advancements in commercial PAT systems have been achieved, most vasculature imaging continues to be performed using a customized system. Therefore, PAT still lacks a standardized protocol for the clinical evaluation of images generated by these systems, but initiatives have been started at multiple levels to establish PAT as a standardized clinical imaging tool in the near future [345].

## Histopathology and immunohistochemistry for validation

Histopathology and immunohistochemistry are the “gold standards” for the evaluation of biological and molecular changes associated with disease pathology or therapeutic response. Regardless of the imaging technology being used for qualitative or quantitative assessment of structural or functional changes associated with vascular response to therapy, it is almost always validated using histology or immunohistochemistry in the research setting. For example, chenages in microvessel density can be examined via immunohistochemistry [346]. This method uses labeled antibodies that bind with biomarkers of the endothelium such as CD31, CD105, and CD34, followed by counting the microvessels under high magnification at a predefined number of hot spots or randomly selected microscopic fields. In a clinical setting, angiogenesis is graded through subjective scoring to determine the correlation with tumor parameters such as malignancy, size, or cancer type [347].

Similar methods can be utilized to examine the morphology of blood vessels in solid tumors. Specific markers for smooth muscle cells and/or pericytes are used to differentiate tumor vasculature from normalized vasculature following anti-angiogenic therapies. α-SMA (smooth muscle actin) is the most common marker used to evaluate vascular morphology. Some other markers such as SM22α [348] have also been used. Common markers for pericytes include α-SMA, high molecular weight melanoma-associated antigen (NG2), desmin, and PDGFR-β. The colocalization of both smooth muscle and pericyte markers in immunohistochemical slides may also be used in the morphological assessment of normalized vasculature [349]. For example, a ratio of pericyte-to-endothelial cells (α-SMA/CD31) can provide information on vessel maturity [19].

Although immunohistochemistry is widely used as a validation technique in a research setting, the lack of a standardized process, a wide range of antibodies used from various suppliers, and differences in manual labeling and counting procedures may introduce significant variations in results from separate research groups. Additionally, histology and immunohistochemistry are performed as an endpoint experiment, which limits their usefulness for monitoring vascular response to treatment regimen. Histological analysis is mostly used as a qualitative measure, and its use as a quantitative analysis is user-dependent and prone to variations among users due to a lack of standardized procedure. Most imaging technology focuses on providing information similar to histological analysis but with minimal invasiveness and in a relatively short time.

## Conclusion and future outlook

In this review, we discussed the different imaging modalities used for characterizing and monitoring vascular changes following cancer onset or anti-angiogenic therapy. The structural and functional changes in vasculature provide crucial insights into cancer disease development as well as therapeutic outcomes. In anti-angiogenic therapy, this information can provide an optimal window for drug delivery to maximize the therapeutic effect. All the imaging techniques presented here have been used for vascular imaging in some capacity, and each modality has its positive and negatives. MRI, CT, and PET can be used for vascular imaging throughout the entire body at the expense of spatial and temporal resolution. Optical and ultrasound imaging modalities are capable of imaging microvasculature at the expense of imaging depth and field of view. Each modality has its own collection of contrast agents that can be used to extend imaging depth, allow for detection of smaller vasculature, or to enable analysis of hemodynamic properties. Moreover, innovative approaches to data acquisition and processing has enabled most imaging modalities to characterize vascular physiology and function, including perfusion, vascular permeability, and blood oxygenation levels. Photoacoustic imaging is an emerging imaging modality capable of evaluating blood oxygen levels without the use of exogenous contrast agents. Multiple imaging modalities also may be combined to overcome respective limitations and to assess the structure and function of blood vessels across multiple scales. There are several treatment strategies capable of altering the morphology and the performance of blood vessels, including radiation and anti-angiogenic drugs. The ability to monitor vascular response longitudinally provides clinicians with the information needed to adjust treatment parameters and maximize therapeutic efficacy. Moreover, imaging the vascular response may be key to identifying the vascular normalization window and exploring new and effective combinations of anti-angiogenic agents and anticancer drugs.

Anti-vascular agents such as bevacizumab, sunitinib, ramucirumab, sorafenib, regorafenib, etc are usually used in combination with chemotherapeutics or immunotherapeutics to improve patient outcomes. Employing one or a combination of the discussed imaging modalities to optimize dosing, identify the optimal delivery window, and determine the sequence of drug administration to maximize the therapeutic benefits would be ideal. However, despite the diagnostic potential of these imaging modalities in vascular assessment, they have yet to be integrated into standard clinical practice to attain such goals. The ultimate challenge in achieving these goals are (i) differences in tumor progression and therapeutic response in animal and human tumors, (ii) inter- and intra-spatial and temporal heterogeneity in tumors resulting in variable therapeutic response within the tumor, and (iii) variable timing and extent of normalization window. More preclinical work to optimize combination regimes, novel biomarkers for identifying normalization phenotype, and better contrast agents, in part, may address the current challenges in the field. Nonetheless, vascular imaging will likely continue to develop as an impactful technique for the diagnosis and treatment of cancer.

## Data Availability

Not applicable.
